# Construction and Application of the Internet Plus Home-Based Case Management Service Model in Health Management

**DOI:** 10.1093/geroni/igaf122.1192

**Published:** 2025-12-31

**Authors:** Wanling Li, Xianmei Cui, Qian Zhang, Huali Miao, Wenjuan Zhu, Linying Wang, Lihua Wu

**Affiliations:** Shanxi Bethune Hospital, Taiyuan, Shanxi, China (People’s Republic); Shanxi Bethune Hospital, Taiyuan, Shanxi, China (People’s Republic); Shanxi Bethune Hospital, Taiyuan, Shanxi, China (People’s Republic); Shanxi Bethune Hospital, Taiyuan, Shanxi, China (People’s Republic); Shanxi Bethune Hospital, Taiyuan, Shanxi, China (People’s Republic); Shanxi Bethune Hospital, Taiyuan, Shanxi, China (People’s Republic); Shanxi Bethune Hospital, Taiyuan, Shanxi, China (People’s Republic)

## Abstract

**Objective:**

China’s existing “Internet Plus Nursing Services” primarily focus on single nursing procedures and are insufficient for the multifaceted health needs of elderly patients. This study incorporates health management into existing services, developing the Precision-Safety-Homogenization (PSH) “Internet Plus Health Management” model and evaluating its effectiveness.

**Methods:**

Conducted in a large general hospital in China, this study provided on-demand health management services for elderly patients.

**Precision:**

Supported by an MDT, comprehensive health assessments were conducted across pre-, in-, and post-hospital phases. The MDT formulated individualized health management plans, which were implemented and monitored by nurses.

**Safety:**

During home visits, nurses assessed patients’ health risks and collaborated with the MDT team to provide coordinated health management, optimizing intervention strategies.

**Homogenization:**

Nurse training and oversight adhered to standardized in-hospital protocols, standardizing service scope, medical supply use, and pricing.

**Results:**

From June 2021 to September 2024, the PSH health management model was applied to 1,244 patients. After six months, key health indicators showed significant improvements: The HbA1c target achievement rate in diabetes patients increased from 45.2% to 72.8% (P = 0.04).The blood pressure control rate in hypertensive patients improved from 50.3% to 78.9% (P = 0.03). The Barthel Index in stroke patients increased from 48.2 to 68.7 (P = 0.01).Lung function (FEV₁%) in COPD patients improved from 58.6% to 72.4% (P = 0.01).A total of 191 adverse events were identified and managed, with 100% patient satisfaction reported.

**Conclusion:**

The PSH health management model effectively identifies diverse health issues in elderly patients, supports structured and evidence-based health management, helps slow disease progression, and enhances patient safety.

